# Effects of Yomogi Tea on Lipid Metabolism in Renal Tubular HK-2 Cells

**DOI:** 10.3390/foods14223817

**Published:** 2025-11-07

**Authors:** Wei Qin, Hsin-Jung Ho, Xun-Zhi Wu, Miki Eguchi, Manami Uchita, Minato Takeuchi, Shu-Ping Hui

**Affiliations:** 1 School of Agriculture, Utsunomiya University, 350, Mine-machi, Utsunomiya 321-8505, Japan; 2 Faculty of Health Sciences, Hokkaido University, Kita-12, Nishi-5, Kita-ku, Sapporo 060-0812, Japan

**Keywords:** yomogi tea, lipid droplets, lipotoxicity, triglycerides, free fatty acids, lipogenesis, lipolysis

## Abstract

Excessive accumulation of lipid droplets (LDs), their dynamics, and lipotoxicity are critical factors in the progression of metabolic disorders, including diabetic nephropathy. This study investigates the effects of yomogi tea (Mugwort tea), specifically its leaf infusion (YL) and powdered infusion (YP), on lipid metabolism in human kidney proximal tubular epithelial HK-2 cells under lipotoxic conditions induced by palmitic acid (PA). Both YL and YP significantly reduced intracellular triglyceride (TG) and free fatty acid (FFA) levels, with YP showing a trend toward greater efficacy. Mechanistic analysis revealed that yomogi tea regulates lipid metabolism by significantly downregulating mRNA expression of FAS and upregulating that of the lipolytic ATGL, while SCD-1 mRNA expression remained largely unchanged. Furthermore, yomogi tea reduced LD size and neutral lipid content, potentially enhancing lipid hydrolysis efficiency and mitigating lipotoxic effects. These findings highlight the potential of yomogi tea as a natural agent for regulating lipid metabolism and reducing lipotoxicity, offering promise for managing lipid metabolism-related disorders.

## 1. Introduction

The rising prevalence of obesity has led to a surge in metabolic disorders such as diabetic nephropathy (DN), atherosclerosis, and non-alcoholic fatty liver disease, which now represent critical global health challenges [1]. Dysregulated lipid metabolism is a key factor driving these conditions, as excessive energy intake in obesity often surpasses the storage capacity of adipose tissue, resulting in ectopic lipid accumulation in non-adipose tissues, including the kidneys [2,3,4,5,6].

Lipid droplets (LDs), as cellular reservoirs for lipid storage, play a vital role in maintaining lipid homeostasis through processes involving lipid synthesis, metabolism, and transport [7]. However, excess accumulation of LDs in renal tubular epithelial cells has been linked to lipotoxicity, contributing to inflammation, reactive oxygen species (ROS) production, and cell death during the progression of DN [8,9,10,11]. These insights have fueled research into innovative therapeutic strategies targeting LD regulation to counteract lipid imbalances and restore lipid homeostasis. Lipid metabolism encompasses a complex network of processes, including synthesis, breakdown, digestion, absorption, and transport, influenced by various factors such as diet, genetic predisposition, and physical activity [2]. Dysregulated lipid metabolism, characterized by imbalances in triglycerides (TG), cholesterol, or free fatty acids (FFAs), is a hallmark of metabolic disorders such as obesity, cardiovascular diseases, and DN. Chronic kidney disease, which affects approximately 10% of the global population and causes over 1.2 million deaths annually, has been linked to ectopic lipid accumulation, identified as a critical driver of disease progression through oxidative stress, lipotoxicity, and LD-associated dysfunction [6,12,13,14,15,16,17,18,19]. Emerging evidence highlights the central role of LDs in cellular lipid dynamics, underscoring their diverse functions as metabolic organelles, their morphological variability, and their potential as indicators of cellular health and metabolism [12,19,20]. Recent studies have demonstrated that natural compounds can modulate LDs’ behavior, offering a promising avenue to address lipid metabolism disorders. Compounds such as berberine, genistein, and resveratrol interact with LD-associated proteins to mitigate lipid accumulation and related toxicity [21]. Furthermore, functional foods and herbal remedies, particularly those rich in bioactive compounds like flavonoids and polyphenols, have shown efficacy in regulating lipid metabolism and inflammatory pathways, with minimal side effects compared to conventional therapies [9,10,11,22]. These findings underscore the potential of dietary interventions in managing metabolic disorders by restoring lipid homeostasis.

Yomogi (Japanese mugwort), commonly known as *Artemisia princeps*, has garnered significant attention for its diverse health benefits, particularly in traditional East Asian medicine. Its bioactive compounds, including flavonoids (e.g., liquiritin and eupafolin) and polyphenolic acids (e.g., chlorogenic acid), exhibit anti-inflammatory, antioxidant, and lipid-lowering properties, making it a promising candidate for functional food applications [9,10,11]. Experimental studies have shown that yomogi extracts can normalize body weight, reduce adipose tissue mass, and influence lipid metabolism in models of obesity and diabetes [10]. Additionally, certain yomogi varieties, such as *Artemisia princeps* Pampanini cv. Sajabal, has demonstrated anti-atherosclerotic properties in animal models by suppressing pro-inflammatory markers and lowering oxidized LDL levels, further supporting their cardiovascular benefits [11]. The lipid-lowering and anti-inflammatory potential of wild yomogi varieties, such as the Mongolian herb TGLG-1, has also been supported by LC-MS analysis, which revealed high concentrations of flavonoids and polysaccharides [9]. Mechanistic studies indicate that yomogi regulates lipid metabolism through multiple pathways, including the inhibition of lipogenesis, reduction in oxidative stress, and modulation of inflammatory responses. Specifically, A. princeps has been shown to suppress inflammation by inhibiting the NF-κB and MAPK signaling pathways; its neutral polysaccharides exhibit anti-complementary activity, suggesting immune-modulating effects, and its extract reduces lipid accumulation during 3T3-L1 adipocyte differentiation, indicating inhibition of adipogenic signaling [23,24,25,26]. Previous reports suggest that these effects may involve modulation of NF-κB inflammatory signaling and Nrf2-mediated antioxidant defenses, as well as AMPK and PPARα/γ pathways activated by Artemisia extracts and related polyphenols. These upstream regulators are functionally connected with lipid-regulatory genes, signaling converges on lipogenic enzymes such as FAS, ACC or SCD-1 [27,28], while inflammatory and antioxidant pathways may influence cellular lipid handling more broadly. These findings emphasize the potential of yomogi as a natural therapeutic agent for managing lipid-related metabolic disorders. Many herbal teas and polyphenol-rich extracts have indeed been reported to influence lipid metabolism [29,30]. However, most of these studies have focused on hepatic or adipocyte models, whereas evidence in renal proximal tubular cells—key sites directly involved in DN-related lipotoxicity—remains limited [31]. Moreover, unlike other teas, yomogi tea contains a distinct chemical profile enriched in sesquiterpenes and unique flavonoid derivatives, suggesting potentially different modes of action compared with more commonly studied teas such as green or black tea [32]. Since the release of these bioactive compounds during infusion can be influenced by preparation methods, particle size has also been shown to affect the extraction efficiency of polyphenols and related metabolites [33,34].

Building on these insights, this study aims to investigate the effects of yomogi tea, especially yomogi tea leaf infusion (YL) and powdered infusion (YP), on lipid metabolism in kidney cells. To achieve this, we utilized human kidney proximal tubular epithelial (HK-2) cells as a model system to examine their ability to regulate lipid storage and breakdown in LDs, attenuate lipid accumulation, and modulate related metabolic pathways. The findings are expected to provide deeper insights into the therapeutic potential of yomogi in addressing lipid metabolism disorders and offer scientific evidence to support its application in functional foods and dietary interventions.

## 2. Materials and Methods

### 2.1. Materials and Preparation

The dried yomogi tea was purchased from a local store is manufactured by Uchida Wakan-Yaku Co., Ltd. (Tokyo, Japan), lot code NXH3932. A portion of the yomogi tea leaves was ground into a fine powder, resulting in yomogi tea powder. All experiments, including both YL and YP, were prepared from this single commercial lot to minimize inter-batch variability.

The weight of tea required for infusion was calculated based on the dry matter (d.m.). Each sample, consisting of 2.5 g of tea leaves or tea powder, was placed in a beaker, and 70 °C water was added to reach a total weight of 250 mL. Both tea leaves/powders were allowed to steep for 5 min, then filtered to obtain the respective tea infusions (YL and YP). The filtrates were stored at −80 °C for further use in experiments, according to the method described by [35], with minor modifications.

### 2.2. Cell Line and Culture Conditions

HK-2 cells are a widely used model of human proximal tubular epithelial cells, which are highly susceptible to lipid accumulation and lipotoxic injury, making them suitable for investigating lipid metabolism in the kidney [6,12,19]. HK-2 cells were purchased from the American Type Culture Collection (ATCC, Manassas, VA, USA). The cells were cultured in Dulbecco’s Modified Eagle Medium (DMEM; Nacalai Tesque, Kyoto, Japan) supplemented with 10% fetal bovine serum (FBS; Biosera, East Sussex, UK) and 1 U/mL penicillin-streptomycin (P/S; Fujifilm Wako, Osaka, Japan). Cultures were maintained at 37 °C in a humidified incubator with 5% CO_2_. Considering the potential variations associated with extended passaging, all experiments were conducted using HK-2 cells within a 10-passage range (approximately between passages 20 and 30), during which the cells maintained stable morphology and proliferation.

### 2.3. Cell Viability Assay

HK-2 cells were seeded into 96-well plates at a density of 4 × 10^3^ cells per well and allowed to adhere for 24 h. Following the initial incubation, the medium was replaced with DMEM containing 1% FBS and 1 U/mL P/S. Palmitic acid (PA) was conjugated to fatty acid-free bovine serum albumin (BSA; Nacalai Tesque, Kyoto, Japan) as described in a previous study [36] with slight modifications. In brief, 500 mM of PA was dissolved in ethanol at 37 °C with continuous mixing until fully dissolved. The solution was then combined with DMEM supplemented with 10% fatty acid-free BSA, achieving a final fatty acid-to-BSA molar ratio of 3.3:1.

Various concentrations of PA (50, 100, 200, and 400 µM), yomogi tea leaf infusion (YL; 12.5, 25, 50, and 100 µg/mL), and yomogi tea powder infusion (YP; 12.5, 25, 50, and 100 µg/mL) were added to the culture medium and incubated for 24 h. Following the treatment period, cell viability was assessed using the cell counting kit-8 (CCK-8; Dojindo, Kumamoto, Japan) in accordance with the manufacturer’s instructions. Absorbance was measured using a microplate reader (xMark Microplate Spectrophotometer, Bio-Rad, Hercules, CA, USA). Based on the preliminary results, concentrations of 200 µM PA and 25, 50, and 100 µg/mL for YL and YP were selected for subsequent experiments to further evaluate their effects. At 200 µM PA, the final culture medium contained 0.02% ethanol and 0.2% BSA. A vehicle control (Control) containing the same BSA and ethanol concentrations but without PA was included.

### 2.4. Cellular Lipid Extraction

HK-2 cells were seeded in a 6-well plate with 1 × 10^5^ cells per well. Following the designated treatments, the culture medium was removed, and the cells were gently washed with phosphate-buffered saline (PBS; Nacalai Tesque) to remove residual media. Cells were then harvested using a cell scraper and subjected to total lipid extraction based on a previously described protocol [7], with slight modifications. Specifically, the cells from each dish were transferred to 1.5 mL microcentrifuge tubes.

For lipid extraction, 900 μL of a solvent mixture comprising MTBE/MeOH/H_2_O in a ratio of 6:2:1 (*v*/*v*) was used. The mixture contained internal standards (IS) (SPLASH™ LIPIDOMIX™ Mass Spec Standard; Gaithersburg, MD, USA), including 400 pmol of TG 15:0/18:1-d7/15:0 and 600 pmol of FFA 19:0, to ensure accurate quantification. Quantification of lipid species was normalized to the signal intensity of the internal standards to correct for extraction efficiency and instrument variability. The extraction process was performed twice to maximize lipid recovery. After extraction, the samples were centrifuged to separate phases, and the organic layer was collected. The collected organic phases were dried under vacuum to remove solvents. The resulting lipid residues were reconstituted in methanol and stored at −80 °C until further analysis.

### 2.5. LC/MS Analysis

Lipidomic analysis and semi-quantitative measurements were performed using a Shimadzu Prominence HPLC system (Shimadzu Corp., Kyoto, Japan) coupled with an LTQ Orbitrap mass spectrometer (Thermo Fisher Scientific Inc., San Jose, CA, USA). The system was operated in both positive and negative electrospray ionization (ESI) modes. Chromatographic separation was achieved under the following conditions [37]: the column used was an Atlantic T3 C18 column (2.1 mm × 150 mm, 3 µm, Waters, Milford, MA, USA); the mobile phases consisted of water with 10 mM ammonium acetate (A), isopropanol (B), and methanol (C); the column oven temperature was maintained at 40 °C; and the flow rate was set at 200 µL/min. Chromatographic separation was performed with a linear gradient elution: 0–15 min, 0% B and 10% C; 15–30 min, 70% B and 30% C; 30–45 min, 100% C. Mass spectrometric parameters were as follows: HR-MS^1^ scan mode utilized a Fourier Transform (FT) analyzer with a resolution of 60,000, and the scan range was m/z 150–1100 in positive mode and m/z 220–1650 in negative mode. For MS^2^ scans, an Ion-trap (IT) analyzer was employed, using collision-induced dissociation (CID) under data-dependent acquisition. The normalized collision energy was set to 35%, and the isolation width was set at 2 Da. Data processing was conducted using Xcalibur 2.3 software (Thermo Fisher Scientific). Lipid quantification was normalized to total protein levels, which were determined using the Pierce BCA Protein Assay Kit (Thermo Fisher Scientific) [7,37]. The results are expressed as relative amounts compared to the control group. The quantity of each lipid species was calculated using the following formula [7,37]:

Amount_analyte_ = Amount_IS_ × Peak area_analyte_/Peak area_IS_

where “Amount_analyte_” represents the calculated amount of the target compound, “Amount_IS_” is the amount of the internal standard added, “Peak area_analyte_” is the LC/MS peak area of the analyte, and “Peak area_IS_” is the peak area of the internal standard.

### 2.6. Oil Red O Staining

To prepare a stock solution, dissolve oil red O powder in isopropanol to make a concentration of 3 mg/mL. A working solution of oil red O in 60% isopropanol was freshly prepared before use by adding deionized water. This mixture was allowed to sit for 10 min to ensure proper mixing and was then filtered using a Millipore Millex-HV 0.45-μm filter (Bedford, MA, USA) to remove any precipitates. For the staining procedure, the culture medium was removed from the cells, and they were washed twice with PBS. The cells were then fixed by adding 10% formalin solution (Sigma Aldrich, St. Louis, MO, USA), followed by incubation for 15 min at room temperature. After fixation, the supernatant was removed, and the cells were washed with PBS and subsequently with a 60% isopropanol solution to enhance permeability. The fixed cells were immersed in the freshly prepared oil red O working solution for 20 min, allowing the dye to stain the neutral lipids effectively. After staining, the cells were rinsed with PBS to remove any unbound dye and were visualized under an Olympus IX71 microscope (Tokyo, Japan) for imaging. Quantification of intracellular lipid content and LDs size in HK-2 cells was performed using ImageJ software (version 1.53), ensuring precise analysis of lipid accumulation. For image analysis, images were converted to 8-bit grayscale, and identical threshold parameters were applied to all groups to identify lipid droplets. The analyze particles function was used to quantify droplet number and total area, and the average radius was calculated from these parameters. All results were normalized to the number of cells per microscopic field to ensure consistency among groups.

### 2.7. mRNA Expression and Reverse Transcription-Quantitative Polymerase Chain Reaction (RT-qPCR)

HK-2 cells were seeded into 6-well plates at a density of 1 × 10^5^ cells per well and cultured for 24 h. After incubation, the cells were treated with PA (200 μM) and YL or YP at concentrations of 25, 50, and 100 µg/mL for another 24 h. Following the treatments, the cells were harvested for further analysis. Total RNA was extracted and purified using the Sepasol^®^-RNA I Super G reagent (Nacalai Tesque), following the manufacturer’s protocol. The concentration and purity of the isolated RNA were assessed using a NanoDrop Microvolume Spectrophotometer (Thermo Fisher Scientific, Wilmington, DE, USA). Reverse transcription was performed to synthesize cDNA using the ReverTra Ace Kit (Toyobo Co., Ltd., Osaka, Japan). Quantitative reverse transcription-polymerase chain reaction (qRT-PCR) was conducted to measure the expression levels of specific target genes. The reactions were performed on a CFX Connect Real-Time PCR Detection System (Bio-Rad) using THUNDERBIRD^®^ SYBR^®^ qPCR Mix (TOYOBO) according to the manufacturer’s protocol. The mRNA expression levels of the following genes were analyzed: glyceraldehyde-3-phosphate dehydrogenase (GAPDH) as the reference gene, adipose triglyceride lipase (ATGL), acetyl-CoA carboxylase (ACC), fatty acid synthase (FAS), and stearoyl-CoA desaturase-1 (SCD-1) as target genes. The list of primers used is provided in Table 1. Thermal cycling conditions included an initial denaturation step at 95 °C for 3 min, followed by 40 cycles of amplification consisting of denaturation at 95 °C for 10 s and annealing/extension at 60 °C for 30 s. Relative mRNA expression levels were normalized to GAPDH and calculated using the 2^−∆∆Cq^ method.

### 2.8. Statistical Analysis

Data are presented as mean ± standard deviation (SD). Statistical significance was determined using one-way ANOVA or two-way ANOVA with Tukey’s post hoc test, conducted in GraphPad Prism 9 (GraphPad Software, Inc., La Jolla, CA, USA). *p* value < 0.05 was considered statistically significant. Experiments were performed in triplicate unless specified otherwise.

## 3. Results

### 3.1. Yomogi Tea Improved Cellular Lipid Content

#### 3.1.1. Cell Viability

**Figure 1:**
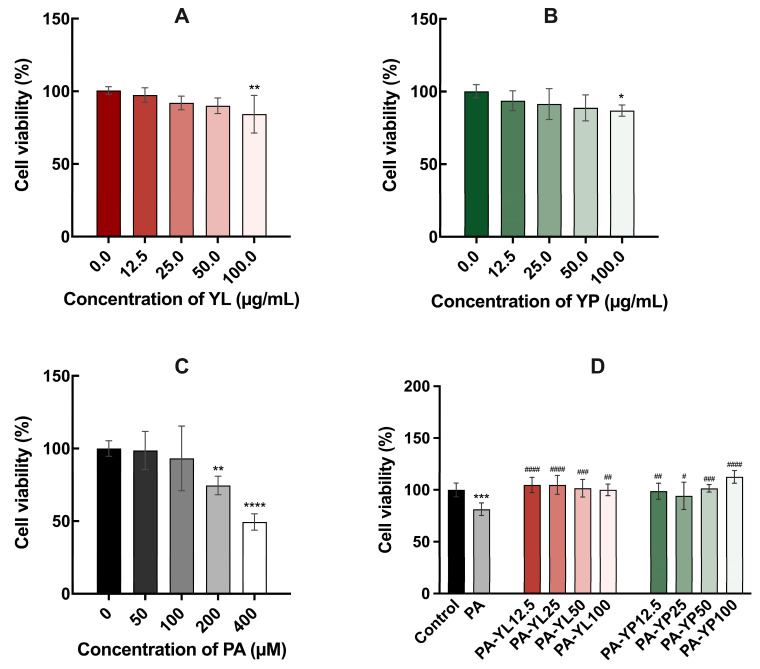
Viability of cells treated with YL (**A**), YP (**B**), PA (**C**), and both PA with YL/YP (**D**). * *p* < 0.05, ** *p* < 0.01, *** *p* < 0.001, **** *p* < 0.0001 vs. control. ^#^ *p* < 0.05, ^##^ *p* < 0.01, ^###^ *p* < 0.001, ^####^ *p* < 0.0001 vs. PA group (n = 6). Control: the vehicle-treated group containing 0.2% BSA and 0.02% ethanol. PA: 200 µM PA; PA + YL12.5: 200 µM PA + 12.5 µg/mL YL; PA + YL25: 200 µM PA + 25 µg/mL YL; PA + YL50: 200 µM PA + 50 µg/mL YL; PA + YL100: 200 µM PA + 100 µg/mL YL; PA + YP12.5: 200 µM PA + 12.5 µg/mL YP; PA + YP25: 200 µM PA + 25 µg/mL YP; PA + YP50: 200 µM PA + 50 µg/mLYP; PA + YP100: 200 µM PA + 100 µg/mL YP.

To determine the appropriate doses for subsequent experiments, the cytotoxic effects of YL and YP on HK-2 cells were first evaluated (Figure 1A,B). The results demonstrated that concentrations below 100 µg/mL of both YL and YP had no significant impact on cell viability, indicating good cellular compatibility. Based on these findings, YL and YP at concentrations of 25, 50, and 100 µg/mL were selected for further investigations. These concentrations were chosen to assess dose-dependent responses while maintaining cell viability. Additionally, the optimal concentration of PA was examined (Figure 1C). Cell viability showed a gradual decline as the concentration of PA increased, indicating a clear dose-dependent cytotoxic response. At 200 µM, cell viability was 74.52 ± 6.38%, a level that allowed sufficient cell activity while successfully inducing lipid overload. Therefore, 200 µM PA was used as the lipid overload inducer in HK-2 cells, creating a condition that resembles the lipid burden observed during the progression of DN. This model was used in subsequent experiments to evaluate the potential protective effects of YL and YP. Additionally, it was confirmed that the co-treatment of YL/YP and PA did not cause additional cytotoxicity to the cells (Figure 1D). Cell viability in some co-treated groups slightly exceeded 100% of the control; however, this increase was within the standard deviation and did not indicate a proliferative effect.

#### 3.1.2. TG and FFA Content

**Figure 2:**
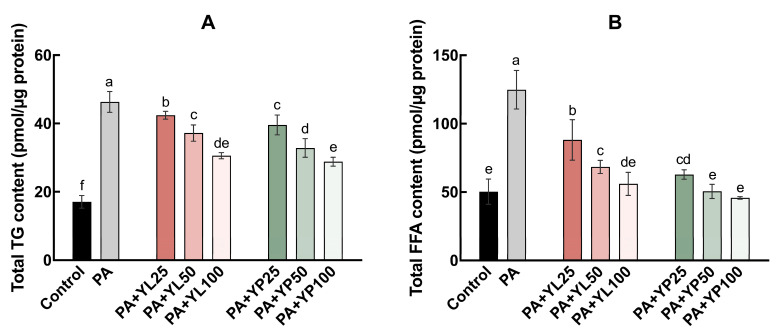
Effects of yomogi tea on cellular lipid accumulation in HK-2 cells. Cellular TG content (**A**). Cellular FFA content (**B**) was measured by LC–MS after 24 h treatment with PA (200 µM) and YL/YP (25, 50, and 100 µg/mL). Control: the vehicle-treated group containing 0.2% BSA and 0.02% ethanol. Data are presented as mean ± SD (n = 4). Values with different letters are significantly different (*p* < 0.05). Different colors indicate different treatment groups.

The effects of yomogi tea on intracellular TG and FFA levels were analyzed using LC/MS. As shown in Figure 2A, treatment with PA significantly increased TG levels in cells, rising from 17.02 ± 1.84 pmol/µg protein in the control group to 46.33 ± 3.03 pmol/µg protein. Upon the addition of YL at varying concentrations, TG levels gradually decreased in a dose-dependent manner, with 100 µg/mL YL reducing TG levels to 30.58 ± 0.90 pmol/µg protein. Similarly, YP treatment also exhibited a dose-dependent effect, with TG levels further reduced to 28.79 ± 1.31 pmol/µg protein at a concentration of 100 µg/mL. Regarding FFA content, the PA-treated group showed a significant increase compared to the control group (124.80 ± 14.11 vs. 50.28 ± 9.26 pmol/µg protein, Figure 2B). Increasing concentrations of YL and YP effectively reduced FFA levels. At 100 µg/mL of YL and YP, FFA levels decreased to 56.02 ± 8.43 and 45.76 ± 0.92 pmol/µg protein, respectively, demonstrating a strong dose-dependent inhibitory effect. The detailed LC–MS results are provided in Table S1 of the Supplementary Materials.

### 3.2. Yomogi Tea Alleviated LDs Accumulation

**Figure 3:**
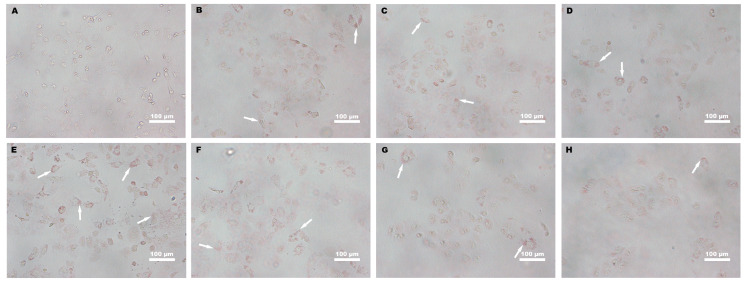
Representative images of oil red O staining showing LD accumulation in HK-2 cells treated with PA (200 µM) and YL/YP (25, 50, and 100 µg/mL) for 24 h. (**A**): Control; (**B**): PA + YL25; (**C**): PA + YL50; (**D**): PA + YL100; (**E**): PA; (**F**): PA + YP25; (**G**): PA + YP50; (**H**): PA + YP100. White arrows indicate representative LDs. Scale bar: 100 µm.

To evaluate the effects of Yomogi tea on lipid deposition, oil red O staining was used to visualize LD accumulation in HK-2 cells. The red-stained particles indicate the presence of neutral lipids within LDs. In the control group (Figure 3A), LD accumulation was barely observed, with almost no visible red staining, indicating a low level of neutral lipids under normal conditions. In contrast, the PA-treated group (Figure 3E) exhibited significant LD accumulation, as evidenced by the intense red staining, confirming the successful induction of a lipotoxic environment by PA. Upon treatment with increasing concentrations of YL and YP, LD accumulation was markedly reduced in a dose-dependent manner (Figure 3B–D,F–H). The intensity of red staining diminished progressively with higher concentrations of yomogi tea, indicating effective mitigation of lipid deposition.

**Figure 4:**
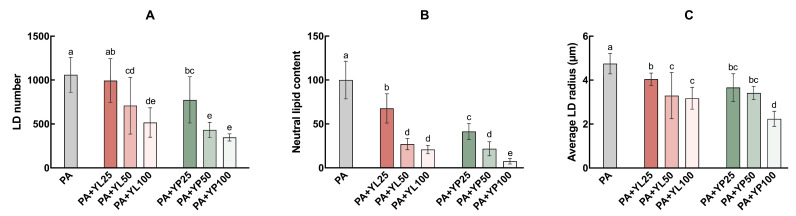
Effect of yomogi tea on LD accumulation in HK-2 cells. Cells were treated with PA (200 µM) and YL/YP (25, 50, and 100 µg/mL) for 24 h. (**A**): LD number. (**B**): Neutral lipid content. (**C**): Average LD radius. Data are presented as mean ± SD (n = 5). Values with different letters are significantly different (*p* < 0.05). All data were normalized to the number of cells per microscopic field. Different colors indicate different treatment groups.

Further quantitative analyses were conducted to evaluate the effects of yomogi tea on LD number, neutral lipid content, and average LD radius (Figure 4). As shown in Figure 4A, the number of LDs in the PA-treated group was significantly higher (1060.00 ± 199.5) compared to the control group. With increasing concentrations of YL, the LD number gradually decreased to 994.80 ± 249.60, 708.80 ± 322.60, and 516.70 ± 167.30 at 25, 50, and 100 µg/mL YL, respectively. A more pronounced reduction was observed in the YP-treated group, where 100 µg/mL YP reduced the LD number to 347.20 ± 40.94, demonstrating a stronger dose-dependent regulatory effect.

Regarding neutral lipid content (Figure 4B), the PA-treated group showed a significant increase, markedly increasing the value to 100.00 ± 21.46, in stark contrast to the control group. Treatment with YL and YP led to a substantial reduction in neutral lipid content in a dose-dependent manner. At 100 µg/mL, YL reduced the content to 20.89 ± 4.86, whereas YP further decreased it to 7.62 ± 3.19, showing a trend toward a stronger lipid-lowering effect.

In terms of average LD radius (Figure 4C), the PA-treated group exhibited the largest LDs with an average radius of 4.75 ± 0.47 µm. Increasing concentrations of YL and YP led to a significant reduction in LD size in a dose-dependent manner. The average LD radius decreased to 3.17 ± 0.50 µm in the YL group at 100 µg/mL, whereas the same concentration of YP further reduced the radius to 2.24 ± 0.34 µm, highlighting YP’s slightly stronger ability to decrease LD size.

### 3.3. Transcriptional Regulation of Lipogenesis and Lipolysis Genes

**Figure 5:**
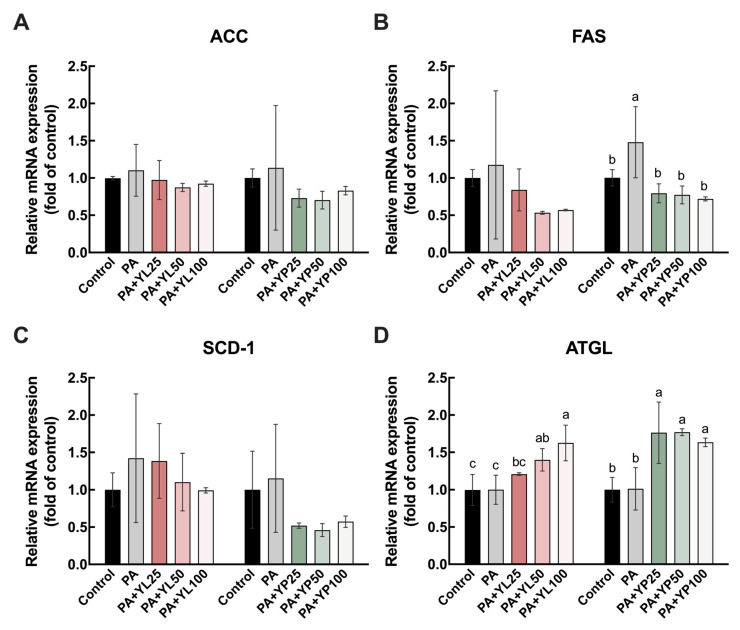
Effects of yomogi tea on the mRNA expression of lipid-metabolism-related genes in HK-2 cells. Cells were treated with PA (200 µM) and YL/YP (25, 50, and 100 µg/mL) for 24 h. Relative mRNA expression levels of ACC (**A**), FAS (**B**), SCD-1 (**C**), and ATGL (**D**) were normalized to GAPDH and are shown as fold change versus control. Data are presented as mean ± SD (n = 3). Values with different letters are significantly different (*p* < 0.05). Different colors indicate different treatment groups.

The expression levels of key genes involved in lipid metabolism, including ACC, FAS, SCD-1, and ATGL, were evaluated to determine the potential regulatory effects of yomogi tea on lipid composition and metabolism in HK-2 cells. The results indicated that ACC, a pivotal enzyme in fatty acid biosynthesis [38], showed no significant changes in expression across the control, PA-treated, and YL/YP-treated groups, suggesting that ACC was not notably affected under the current experimental conditions (Figure 5A). In contrast, FAS, another critical enzyme in fatty acid synthesis [38], demonstrated a significant reduction in expression with increasing concentrations of both YL and YP. The suppression of FAS was particularly pronounced in the YP-treated groups, where the expression levels were significantly lower than in the PA-treated group, indicating a strong inhibitory effect on fatty acid synthesis (Figure 5B). For SCD-1, an enzyme involved in the desaturation of FFA [38], a slight upregulation was observed in the PA-treated group. However, no significant inhibitory effects were observed in the YL and YP treatment groups, suggesting that SCD-1 expression was not substantially influenced by yomogi tea under these experimental conditions (Figure 5C). ATGL, a rate-limiting enzyme responsible for triglyceride hydrolysis in LDs, displayed a marked and dose-dependent increase in expression in response to YL and YP treatment. The upregulation of ATGL was most evident in the 100 µg/mL YP-treated group, where expression levels were significantly higher than those in the PA-only group, indicating enhanced TG breakdown and lipid droplet hydrolysis (Figure 5D).

## 4. Discussion

Ectopic lipid accumulation is a pathological condition that disrupts lipid homeostasis, leading to severe metabolic disorders. In the kidneys, ectopic lipid accumulation in the tubular region induces lipotoxic effects, which have been recognized as a novel mechanism driving the progression of DN [19]. Recent studies suggest that regulating lipid metabolism can effectively alleviate diabetes-related complications, including DN [39,40]. Herbal beverages have garnered increasing attention due to their natural origin, potential health benefits, and low risk of side effects [41,42]. This study focuses on yomogi tea, systematically exploring the regulatory effects of YL and YP on lipid metabolism at the cellular level, along with its potential protective mechanisms. Both YL and YP demonstrated significant effects in reducing lipid accumulation, albeit with differences in efficacy. LC/MS analysis revealed that YP was more effective than YL in lowering total TG and FFA levels (Figure 2A,B), which may be attributed to the enhanced release efficiency of bioactive components in the powdered form [43]. The smaller particle size and larger surface area of YP likely facilitated a higher release of active compounds during the extraction process, thereby enhancing its regulatory capacity on lipid metabolism. Previous reports have confirmed that yomogi tea contains measurable levels of polyphenols and flavonoid derivatives, although generally lower than those of green tea [35]. This supports the notion that the differences observed between YL and YP may be linked to the extraction efficiency of these bioactive constituents. As an optimized form, YP holds greater potential for practical applications. Excessive TG storage in non-adipose cells can induce lipotoxicity (Figure 1C), impairing their normal function. The ability of yomogi tea to significantly reduce TG and FFA accumulation (Figure 2A,B) suggests that its active compounds may alleviate lipid overload by inhibiting lipid synthesis or promoting lipid breakdown. This effect likely mitigates the harmful impact of LDs on cellular function.

The multidimensional regulatory effects of yomogi tea on LD morphology also warrant attention. Oil red O staining results demonstrated that yomogi tea significantly reduced the size and number of LDs induced by PA treatment (Figure 3 and Figure 4). Smaller LDs, by providing a larger surface area, can effectively enhance lipase-mediated lipid breakdown, whereas larger LDs often lead to impaired TG metabolism, disrupted lipid utilization, and reduced energy production due to insufficient breakdown [7,12,44]. This study revealed that yomogi tea significantly decreased the average diameter of LDs (Figure 4), preventing the formation of oversized LDs and supporting the maintenance of normal TG and FFA metabolic levels. Additionally, oil red O staining further indicated that yomogi tea dose-dependently reduced the neutral lipid content within the core of LDs, reinforcing its multilayered impact on lipid metabolism. YP at high concentrations (100 µg/mL) demonstrated a more pronounced effect on reducing LD size compared to YL (Figure 3 and Figure 4), suggesting that YP possesses enhanced lipid breakdown efficiency or stronger regulatory capacity in lipid metabolism, making it more effective in mitigating lipotoxicity.

The potential molecular mechanisms by which yomogi tea regulates lipid metabolism were elucidated by analyzing the mRNA expression levels of lipogenic and lipolytic genes. Notably, its powdered form (YP) demonstrated a remarkable ability to downregulate lipogenic gene FAS and upregulate the lipolytic gene ATGL. In contrast, ACC and SCD-1 expression was not significantly affected by yomogi tea under the current experimental conditions (Figure 5A,C). This indicates that the primary lipogenesis-related target of yomogi tea is FAS, leading to suppression of excessive fatty acid synthesis. Such regulation is in line with findings from other natural compounds. For instance, arctiin, a lignan-type polyphenolic compound, significantly decreased lipid accumulation in the liver of mice fed a high-fat diet by inhibiting the expression of FAS and SCD-1 [45], suggesting that different natural products may converge on overlapping but not identical lipid regulatory pathways.

The role of yomogi tea in promoting lipolysis is also noteworthy: the expression of ATGL, a rate-limiting enzyme in lipid droplet breakdown, was significantly upregulated in both YL- and YP-treated groups, with the most pronounced effect observed in the YP group (Figure 5D). The upregulation of ATGL facilitates the accelerated hydrolysis of TG, reducing lipid droplet accumulation, as confirmed by LC/MS-based cellular lipidomic analysis (Figure 2). Compared with single-compound interventions such as arctiin, yomogi tea is consumed as a beverage, and the cellular readout reflects combined exposure to multiple constituents at physiologically achievable levels. This complex yet mild exposure profile may coordinate the concurrent downregulation of lipogenesis and promotion of lipolysis observed here, offering a translationally practical complement to single-compound strategies. Studies have reported that the lipophagy pathway may work in concert with lipolysis, where the fusion of autophagosomes and lysosomes further degrades LDs, releasing FFA for energy production [20]. Impaired lipophagy has been shown to exacerbate ectopic lipid deposition in DN and aggravate tubular cell damage [19]. Some previous studies have suggested that polyphenol-rich herbal extracts can modulate lipid metabolism through the autophagy–lipophagy pathway. For example, resveratrol and epigallocatechin gallate have been shown to promote lipophagy and thereby reduce lipid accumulation in renal and hepatic models [46,47]. Therefore, yomogi tea might alleviate lipotoxicity-induced cellular damage by regulating lipolytic gene expression and enhancing the lipophagy pathway. This potential mechanism warrants further investigation. In addition, lipid accumulation in lipotoxic environments is often accompanied by increased oxidative stress. Although this study did not directly measure ROS levels, previous research has shown that natural polyphenolic compounds, such as catechins, exhibit significant protective effects by scavenging ROS and enhancing antioxidant enzyme activity [45,48]. The active components of yomogi tea may possess similar antioxidant properties, indirectly mitigating oxidative stress induced by lipotoxicity. In previous studies, we found that although the total polyphenol content in yomogi tea is not as high as that in green tea, it still has a certain degree of antioxidant activity [35].

Despite these promising findings, several limitations should be acknowledged. This study was restricted to an in vitro model using HK-2 cells, which provides an initial platform for mechanistic exploration but cannot fully reflect the complex physiological environment of the kidney. Future investigations employing animal models or clinical studies will be essential to validate the translational significance of our findings. Moreover, lipotoxicity was induced by PA alone, which represents a simplified condition compared to the diverse lipid species and signaling factors contributing to lipotoxic stress in vivo. Nevertheless, PA-induced lipid overload is a widely accepted and established model for investigating lipid accumulation and lipotoxic injury in renal tubular cells, and has been extensively applied in previous studies [8,12]. By adopting this model, we aimed to provide a controlled and reproducible system to evaluate the protective effects of yomogi tea, while acknowledging the need for more complex models in future work. In addition, our mechanistic analysis was limited to a small set of lipid synthesis and lipolysis genes. Although these markers provided useful insights, we recognize that a more comprehensive assessment, including β-oxidation, mitochondrial function, and autophagy, as well as protein-level validation, is warranted. Investigations will also evaluate oxidative stress pathways, including ROS generation, antioxidant defenses, and biochemical compounds of yomogi to clarify their contribution to the observed protective effects. These aspects will be the focus of our future work.

## 5. Conclusions

This study highlights the potential of yomogi tea as a natural agent for regulating lipid metabolism and mitigating lipotoxicity-induced cellular damage. Both YL and YP significantly reduced TG and FFA levels, with YP showing a trend toward greater efficacy. Yomogi tea exerts its effects through a dual regulatory mechanism involving the suppression of lipid synthesis and the promotion of lipid breakdown. It downregulates FAS expression while upregulating ATGL, thereby facilitating TG hydrolysis and reducing LD accumulation. These changes may enhance lipid turnover efficiency and prevent metabolic disturbances associated with excessive LD formation. Overall, yomogi tea demonstrates a multifaceted role in lipid metabolism regulation, providing a natural, effective, and safe approach for managing lipid metabolism–related disorders such as metabolic syndrome and diabetes. Future studies should further elucidate its molecular mechanisms and evaluate its therapeutic potential in in vivo models and clinical settings.

## Figures and Tables

**Table 1:** Sequences of primers for quantitative real-time PCR.

Target	Primer Sequence (5′-3′)	Accession Number
GAPDH	F: ACCCAGAAGACTGTGGATGG R: CAGTGAGCTTCCCGTTCAG	NM_002046.7
ACC	F: GGAACATCCCTACGCTAAACAG R: CTGACAAGGTGGAGTGAATGAG	NM_198838.2
FAS	F: ACAGGGACAACCTGGAGTTCT R: CTGTGGTCCCACTTGATGAGT	NM_004104.5
SCD-1	F: GGGAGTGTGTCTGCTGAGTAAG R: GCAAGGACTGTTAGAAATCCG	NM_005063.5
ATGL	F: AGACAAACTGCCACTCTATGAG R: GAACTGGATGCTGGTGTTG	NM_020376.4

## Data Availability

The original contributions presented in this study are included in the article/Supplementary Materials. Further inquiries can be directed to the corresponding author.

## Supplementary Materials

The following supporting information can be downloaded at: https://www.mdpi.com/article/10.3390/foods14223817/s1, Table S1: Content of TG and FFA species by LC/MS (pmol/mg protein).

## References

[1] Heindel J.J., Blumberg B., Cave M., Machtinger R., Mantovani A., Mendez M.A., Nadal A., Palanza P., Panzica G., Sargis R. (2017). Metabolism disrupting chemicals and metabolic disorders. Reprod. Toxicol..

[2] Hou C., Zhang W.M., Li J.K., Du L., Lv O., Zhao S.J., Li J. (2019). Beneficial Effects of Pomegranate on Lipid Metabolism in Metabolic Disorders. Mol. Nutr. Food Res..

[3] Moghadasian M.H., Kaur R., Kostal K., Joshi A.A., Molaei M., Le K., Fischer G., Bonomini F., Favero G., Rezzani R. (2019). Anti-Atherosclerotic Properties of Wild Rice in Low-Density Lipoprotein Receptor Knockout Mice: The Gut Microbiome, Cytokines, and Metabolomics Study. Nutrients.

[4] Whaley-Connell A., Sowers J.R. (2017). Obesity and kidney disease: From population to basic science and the search for new therapeutic targets. Kidney Int..

[5] Kotlyarov S., Bulgakov A. (2021). Lipid Metabolism Disorders in the Comorbid Course of Nonalcoholic Fatty Liver Disease and Chronic Obstructive Pulmonary Disease. Cells.

[6] Guebre-Egziabher F., Alix P.M., Koppe L., Pelletier C.C., Kalbacher E., Fouque D., Soulage C.O. (2013). Ectopic lipid accumulation: A potential cause for metabolic disturbances and a contributor to the alteration of kidney function. Biochimie.

[7] Wu X.Z., Chen Z., Wu Y., Chen Y.F., Jia J.P., Shen N.Q., Chiba H., Hui S.P. (2022). Flazin as a Lipid Droplet Regulator against Lipid Disorders. Nutrients.

[8] Thongnak L., Pongchaidecha A., Lungkaphin A. (2020). Renal Lipid Metabolism and Lipotoxicity in Diabetes. Am. J. Med. Sci..

[9] Jiale Chang Y.J. (2023). Baoxin Zhang, Xiangyu Kong, Jirigala Ariben, Ting Hao, Siqin Li, Xing Wang, Study on Lipid-Lowering and Anti-Inflammatory Effects of Wild Mugwort (Mongolian Medicine TGLG-1) Based on LC-MS. Adv. Clin. Med..

[10] Yamamoto N., Kanemoto Y., Ueda M., Kawasaki K., Fukuda I., Ashida H. (2011). Anti-obesity and anti-diabetic effects of ethanol extract of Artemisia princeps in C57BL/6 mice fed a high-fat diet. Food Funct..

[11] Han J.M., Kim M.J., Baek S.H., An S., Jin Y.Y., Chung H.G., Baek N.I., Choi M.S., Lee K.T., Jeong T.S. (2009). Antiatherosclerotic effects of Artemisia princeps Pampanini cv. Sajabal in LDL receptor deficient mice. J. Agric. Food Chem..

[12] Chen Z., Shrestha R., Yang X.Y., Wu X.Z., Jia J.P., Chiba H., Hui S.P. (2022). Oxidative Stress and Lipid Dysregulation in Lipid Droplets: A Connection to Chronic Kidney Disease Revealed in Human Kidney Cells. Antioxidants.

[13] Luyckx V.A., Tonelli M., Stanifer J.W. (2018). The global burden of kidney disease and the sustainable development goals. Bull. World Health Organ..

[14] GBD Chronic Kidney Disease Collaboration (2020). Global, regional, and national burden of chronic kidney disease, 1990–2017: A systematic analysis for the Global Burden of Disease Study 2017. Lancet.

[15] Hsu C.N., Hou C.Y., Chang C., Tain Y.L. (2022). Resveratrol Butyrate Ester Protects Adenine-Treated Rats against Hypertension and Kidney Disease by Regulating the Gut-Kidney Axis. Antioxidants.

[16] Fogo A.B. (2007). Mechanisms of progression of chronic kidney disease. Pediatr. Nephrol..

[17] Rapa S.F., Di Iorio B.R., Campiglia P., Heidland A., Marzocco S. (2020). Inflammation and Oxidative Stress in Chronic Kidney Disease-Potential Therapeutic Role of Minerals, Vitamins and Plant-Derived Metabolites. Int. J. Mol. Sci..

[18] Gai Z.B., Wang T.Q., Visentin M., Kullak-Ublick G.A., Fu X.J., Wang Z.G. (2019). Lipid Accumulation and Chronic Kidney Disease. Nutrients.

[19] Han Y.C., Xiong S., Zhao H., Yang S.K., Yang M., Zhu X.J., Jiang N., Xiong X.F., Gao P., Wei L. (2021). Lipophagy deficiency exacerbates ectopic lipid accumulation and tubular cells injury in diabetic nephropathy. Cell Death Dis..

[20] Olzmann J.A., Carvalho P. (2019). Dynamics and functions of lipid droplets. Nat. Rev. Mol. Cell Biol..

[21] Jiang X.Y., Wang H.Z., Nie K.X., Gao Y., Chen S., Tang Y.H., Wang Z., Su H., Dong H. (2024). Targeting lipid droplets and lipid droplet-associated proteins: A new perspective on natural compounds against metabolic diseases. Chin. Med..

[22] Sandner G., König A., Wallner M., Weghuber J. (2020). Functional foods—Dietary or herbal products on obesity: Application of selected bioactive compounds to target lipid metabolism. Curr. Opin. Food Sci..

[23] Trinh H.T., Lee I.A., Hyun Y.J., Kim D.H. (2011). Artemisia princeps Pamp. Essential oil and its constituents eucalyptol and α-terpineol ameliorate bacterial vaginosis and vulvovaginal candidiasis in mice by inhibiting bacterial growth and NF-κB activation. Planta Med..

[24] Joh E.-h., Trinh H.-t., Han M.J., Kim D.-H. (2010). Anti-Inflammatory Effect of Fermented Artemisia princeps Pamp in Mice. Biomol. Ther..

[25] Zhao Q.-C., Kiyohara H., Yamada H. (1993). Anti-complementary neutral polysaccharides from leaves of Artemisia princeps. Phytochemistry.

[26] Kim M.-J., Park M., Jeong M.K., Yeo J., Cho W.-I., Chang P.-S., Chung J.-H., Lee J. (2010). Radical scavenging activity and anti-obesity effects in 3T3-L1 preadipocyte differentiation of Ssuk (Artemisia princeps Pamp.) extract. Food Sci. Biotechnol..

[27] Lee J., Narayan V.P., Hong E.Y., Whang W.K., Park T. (2017). Artemisia Iwayomogi Extract Attenuates High-Fat Diet-Induced Hypertriglyceridemia in Mice: Potential Involvement of the Adiponectin-AMPK Pathway and Very Low Density Lipoprotein Assembly in the Liver. Int. J. Mol. Sci..

[28] Oh J.H., Karadeniz F., Jang M.-S., Kim H., Seo Y., Kong C.-S. (2021). Loliolide from Artemisia princeps Suppresses Adipogenesis in Human Bone Marrow-Derived Mesenchymal Stromal Cells via Activation of AMPK and Wnt/β-catenin Pathways. Appl. Sci..

[29] Yan Z., Zhong Y., Duan Y., Chen Q., Li F. (2020). Antioxidant mechanism of tea polyphenols and its impact on health benefits. Anim. Nutr..

[30] Abiri B., Amini S., Hejazi M., Hosseinpanah F., Zarghi A., Abbaspour F., Valizadeh M. (2023). Tea’s anti-obesity properties, cardiometabolic health-promoting potentials, bioactive compounds, and adverse effects: A review focusing on white and green teas. Food Sci. Nutr..

[31] Ren L., Cui H., Wang Y., Ju F., Cai Y., Gang X., Wang G. (2023). The role of lipotoxicity in kidney disease: From molecular mechanisms to therapeutic prospects. Biomed. Pharmacother..

[32] Sharifi-Rad J., Herrera-Bravo J., Semwal P., Painuli S., Badoni H., Ezzat S.M., Farid M.M., Merghany R.M., Aborehab N.M., Salem M.A. (2022). *Artemisia* spp.: An Update on Its Chemical Composition, Pharmacological and Toxicological Profiles. Oxid. Med. Cell Longev..

[33] Makanjuola S.A. (2017). Influence of particle size and extraction solvent on antioxidant properties of extracts of tea, ginger, and tea–ginger blend. Food Sci. Nutr..

[34] Shaukat H., Ali A., Zhang Y., Ahmad A., Riaz S., Khan A., Mehany T., Qin H. (2023). Tea polyphenols: Extraction techniques and its potency as a nutraceutical. Front. Sustain. Food Syst..

[35] Qin W., Hu F.F., Hui S.P. (2025). Comparative Study of Total Polyphenol Content and Antioxidant Activity of Yomogi Tea and Green Tea during Simulated In Vitro Gastrointestinal Digestion. ACS Food Sci. Technol..

[36] Ho H.J., Aoki N., Wu Y.J., Gao M.C., Sekine K., Sakurai T., Chiba H., Watanabe H., Watanabe M., Hui S.P. (2023). A Pacific Oyster-Derived Antioxidant, DHMBA, Protects Renal Tubular HK-2 Cells against Oxidative Stress via Reduction of Mitochondrial ROS Production and Fragmentation. Int. J. Mol. Sci..

[37] Wu Y., Chen Z., Fuda H., Tsukui T., Wu X., Shen N., Saito N., Chiba H., Hui S.-P. (2021). Oxidative Stress Linked Organ Lipid Hydroperoxidation and Dysregulation in Mouse Model of Nonalcoholic Steatohepatitis: Revealed by Lipidomic Profiling of Liver and Kidney. Antioxidants.

[38] Zhang H.J., Gao X., Guo X.F., Li K.L., Li S., Sinclair A.J., Li D. (2021). Effects of dietary eicosapentaenoic acid and docosahexaenoic acid supplementation on metabolic syndrome: A systematic review and meta-analysis of data from 33 randomized controlled trials. Clin. Nutr..

[39] Testa R., Bonfigli A.R., Genovese S., De Nigris V., Ceriello A. (2016). The Possible Role of Flavonoids in the Prevention of Diabetic Complications. Nutrients.

[40] Den Hartogh D.J., Gabriel A., Tsiani E. (2020). Antidiabetic Properties of Curcumin II: Evidence from In Vivo Studies. Nutrients.

[41] Poswal F.S., Russell G., Mackonochie M., MacLennan E., Adukwu E.C., Rolfe V. (2019). Herbal Teas and their Health Benefits: A Scoping Review. Plant Food Hum. Nutr..

[42] Shaik M.I., Hamdi I.H., Sarbon N.M. (2023). A comprehensive review on traditional herbal drinks: Physicochemical, phytochemicals and pharmacology properties. Food Chem. Adv..

[43] Qin W., Yamada R., Araki T., Ogawa Y. (2022). Changes in Morphological and Functional Characteristics of Tea Leaves During Japanese Green Tea (Sencha) Manufacturing Process. Food Bioprocess Technol..

[44] Yang H.Y., Galea A., Sytnyk V., Crossley M. (2012). Controlling the size of lipid droplets: Lipid and protein factors. Curr. Opin. Cell Biol..

[45] Cheng C., Li Z.Z., Zhao X., Liao C.L., Quan J., Bode A.M., Cao Y., Luo X.J. (2020). Natural alkaloid and polyphenol compounds targeting lipid metabolism: Treatment implications in metabolic diseases. Eur. J. Pharmacol..

[46] Lin L., Zeng L., Liu A., Yuan D., Peng Y., Zhang S., Li Y., Chen J., Xiao W., Gong Z. (2021). Role of Epigallocatechin Gallate in Glucose, Lipid, and Protein Metabolism and L-Theanine in the Metabolism-Regulatory Effects of Epigallocatechin Gallate. Nutrients.

[47] Zhou F., Deng S., Luo Y., Liu Z., Liu C. (2025). Research Progress on the Protective Effect of Green Tea Polyphenol (-)-Epigallocatechin-3-Gallate (EGCG) on the Liver. Nutrients.

[48] Wu X.Z., Ho H.J., Eguchi M., Chen Z., Chiba H., Hui S.P. (2023). Flazin improves mitochondrial dynamics in renal tubular epithelial cells under oxidative stress. Food Biosci..

